# A Retrospective Cohort Study: Safety and Effectiveness of Elbasvir/Grazoprevir ± Ribavirin Compared With Ombitasvir/Paritaprevir/Ritonavir/Dasabuvir ± Ribavirin in Patients With Chronic Hepatitis C Genotype 1 Infection

**DOI:** 10.3389/fphar.2021.640317

**Published:** 2021-09-09

**Authors:** Hsuan-Yu Hung, Chung-Yu Chen, Yi-Hsiang Liao

**Affiliations:** ^1^ Department of Pharmacy, Ditmanson Medical Foundation Chia-Yi Christian Hospital, Chiayi, Taiwan; ^2^ Master Program in Clinical Pharmacy, School of Pharmacy, Kaohsiung Medical University, Kaohsiung, Taiwan; ^3^ Department of Pharmacy, Kaohsiung Medical University Hospital, Kaohsiung, Taiwan; ^4^ Department of Medical Research, Kaohsiung Medical University Hospital, Kaohsiung, Taiwan; ^5^ Chinese Medical Department, Park One International Hospital, Kaohsiung, Taiwan

**Keywords:** hepatitis C virus, viekirax, zepatier, drug-induced liver injury, DILI

## Abstract

**Background:** The direct-acting antiviral (DAA) agents are widely used to treat hepatitis C virus (HCV) genotype (GT) 1 infection, while it may cause severe liver damage. The objectives of the study were to evaluate the incidence of drug-induced liver injury (DILI), sustained virologic response at post-treatment week 12 (SVR12), and recurrence rates in HCV GT 1 infection.

**Methods:** This was a retrospective cohort study that included patients diagnosed with HCV GT 1 infection, who had received intervention and treatment with elbasvir/grazoprevir (EBR/GZR) ± ribavirin (RBV) versus ombitasvir/paritaprevir/ritonavir (OBV/PTV/r) + dasabuvir ± RBV (as control group) for 12 or 24 weeks at a regional hospital in southern Taiwan between April 2016 and August 2018. The primary outcome of the study was to compare the incidence rate ratio (IRR) of DILI *via* Poisson regression, and the secondary outcome was to evaluate the effectiveness of two treatment regimens expressed as a percentage.

**Results:** The study included 149 patients in the control group and 105 patients in the intervention group of which 99.33 and 98.1% patients, respectively, achieved SVR12. In the control group, one patient experienced relapse, whereas in the intervention group, two patients relapsed. Furthermore, in the control group, a total of nine patients developed DILI as determined during follow-up care. Of these patients, three were 55–84 years old. In the intervention group, six patients developed DILI. The IRR of DILI caused by EBR/GZR treatment was 2.84 times higher than that caused by the OBV/PTV/r treatment regimen.

**Conclusion:** There was no significant difference between the studied DAA regimens regarding the incidence of DILI and effectiveness during the treatment. DILI occurrence during therapy did not affect the cure rate of medication. The present study results can provide reference data for drug selection among patients with HCV.

**Trial registration:** The study was approved by DMF-CYCH (CYCH IRB No: 2018067).

## Introduction

Ombitasvir (OBV), an antiviral agent used for the treatment of hepatitis C virus (HCV) infection, acts as an inhibitor of the non-structural protein 5A (NS5A) inhibitor. Furthermore, paritaprevir (PTV) acts as an HCV NS3/4A protease inhibitor, and ritonavir acts as a CYP3A inhibitor. When these drugs are administered in combination as the OBV/PTV/r regimen, they serve as effective direct-acting antiviral (DAA) agents for the treatment of HCV infection. Furthermore, ribavirin (RBV) in combination with daclatasvir (DSV), an HCV non-nucleoside NS5B palm domain polymerase inhibitor, can be used for the treatment of adult patients with chronic HCV genotype (GT) 1 or 4 infection (Product Information, 2015). Elbasvir (EBR) is an HCV NS5A inhibitor, and grazoprevir (GZR) is an HCV NS3/4A protease inhibitor; they are used as a novel fixed-dose combination product for the treatment of adults with HCV GT 1 or 4 infection (Product Information, 2016). RBV can also be added based on the severity of cirrhosis in patients.

However, the Food and Drug Administration (FDA) issued a drug safety warning on October 22, 2015, regarding the OBV/PTV/r combination product, which may cause severe liver damage, especially in patients with end-stage liver disease (FDA, 2015); moreover, a warning was issued by the FDA in August 2019 against Zepatier after patients exhibited signs and symptoms of moderate-to-severe liver impairment (Child–Pugh class B or C) after its consumption (FDA, 2019).

DAA agents were first introduced in Taiwan in January 2017, and there is limited information regarding the effectiveness and safety of DILI, when EBR/GZR was compared with those of OBV/PTV/r. Furthermore, OBV/PTV/r and EBR/GZR have been associated with DILI. DILI causes various degrees of organ dysfunction based on the extent of exposure to medication or a non-infectious toxic agent. The first-line treatment against DILI is discontinuation of the medication that has triggered the adverse event (Marrone et al., 2017).

Because OBV/PTV/r and EBR/GZR are important medications used in the treatment of HCV infection and there is limited evidence and experience regarding their effectiveness and use, respectively, these regimens must be further investigated. The purpose of this study was to compare the effectiveness of OBV/PTV/r + DSV and EBR/GZR as well as the risk of DILI due to these in patients with chronic HCV GT 1 infection based on the data from a single hospital.

## Materials and Methods

### Patient and Public Involvement

Patients or the public were not involved in the design, conduct, reporting, or dissemination plans of our research.

### Study Design

This is a retrospective cohort study that was approved by the Institutional Review Board (IRB) of the DMF-CYCH (CYCH IRB No: 2018067), which waived the requirement for written informed consent. Data were collected from a regional hospital in southern Taiwan. Patients diagnosed with HCV GT 1 infection between April 1, 2016, and August 31, 2018, who were treated with EBR/GZR or OBV/PTV/r + DSV for >6 weeks were included in this study, and were first time prescribed EBR/GZR or OBV/PTV/r + DSV as index date. Advanced treatment with RBV was added to both the regimens based on the severity of cirrhosis.

### Study Population

Hospital records of eligible patients from April 1, 2016, to August 31, 2018, were retrieved. Eligible patients included those who were diagnosed with chronic HCV GT 1 infection and treated for >6 weeks with OBV/PTV/r (12.5 mg/75 mg/50 mg) twice a day, DSV (250 mg) twice a day with or without RBV (body weight <75 kg for 1,000 or 1,200 mg/day for body weight >75 kg), or EBR/GZR (100 mg/50 mg) once daily with or without RBV depending on patients’ health condition.

We excluded patients diagnosed with non-HCV GT 1 infection and treated with other medications or treated for <6 weeks: those undergoing peritoneal dialysis or hemodialysis, or those with a history of liver or kidney transplantation. We also considered the potential drug–drug interactions; thus, we excluded patients with hepatitis B virus (HBV) infection and human immunodeficiency virus infection to minimize bias and reduce interference. Because the number and timing of patients’ return visits vary, the weeks with the largest number of samplings were selected for intersection. Finally, test values at weeks 1, 3, 5, and 9 were considered; however, these would have been further ruled out if there were no test values at these weeks.

### Outcomes

The goals of the study were to determine the safety and efficacy of the HCV regimens. The viral load was detected by measuring the amount of plasma HCV RNA virus post-treatment at week 12 to assess the effectiveness of the treatment regimen. If the plasma HCV RNA level measured *via* quantitative tests was below the lower limit of quantitation (LLOQ) or <15 IU/ml, the virus was considered undetectable in the blood, indicating that the patient had achieved sustained virologic response at post-treatment week 12 (SVR12). Virologic failure was defined as the failure of plasma HCV RNA level when declined by > 2 logs after 4 weeks of treatment or when the blood HCV RNA level remained higher than LLOQ within 6 weeks of treatment. Patients were considered to have experienced relapse if the HCV RNA virus was detected again after drug discontinuation or after achieving SVR12, with the blood HCV RNA level being lower than LLOQ after the completion of the treatment course (Roth et al., 2015).

In this study, DILI was considered the key safety outcome. The DILI adverse events (AEs) were defined as follows: 1) alanine aminotransferase (ALT) > 5 times upper limit of normal (ULN), 2) aspartate aminotransferase (AST) > 5 times ULN, 3) ALT or AST > 3 times ULN with T-Bil > 2 times ULN, 4) ALT or AST > 3 times ULN with international normalized ratio (INR) > 1.5, and 5) T-Bil > 2 times ULN with INR > 1.5. All cases were traced until February 28, 2019, with the index date entered into study analysis. Each case was followed up until the first occurrence of one of the outcome measures or until the end of the follow-up period. All cases in which none of the outcomes had occurred by the end of follow-up were defined as censored.

### Statistical Analysis

Categorical variables were presented as percentages (%), and the chi-square test was used to determine significant differences. Risk ratio (RR) was calculated using Fisher’s exact test. Mean and SD were used to describe continuous variables, and t-test was used to determine between-group significant differences. A repeated measures analysis of variance was used to compare the laboratory data between two groups.

Furthermore, a sensitivity analysis was determined based on redefining liver injury according to the guidelines in Taiwan. Liver injury was redefined based on the following parameters: 1) ALT >3 times ULN, 2) alkaline phosphatase (ALP) > 2 times ULN, or 3) T-Bil > 2 times ULN with increased ALT or ALP levels. DILI was defined according to the changes in the standard values of ALT and AST by 3–5 times ULN.

Poisson regression was used to calculate the incidence rate ratio (IRR). Significance level was set at *p* < 0.05, and 95% confidence interval (CI) was used to present the value interval. All data processing and statistical analyses were performed using SAS software version 9.4 (SAS Institute, Cary, NC, United States).

## Results

### Patient Characteristics

**FIGURE 1:**
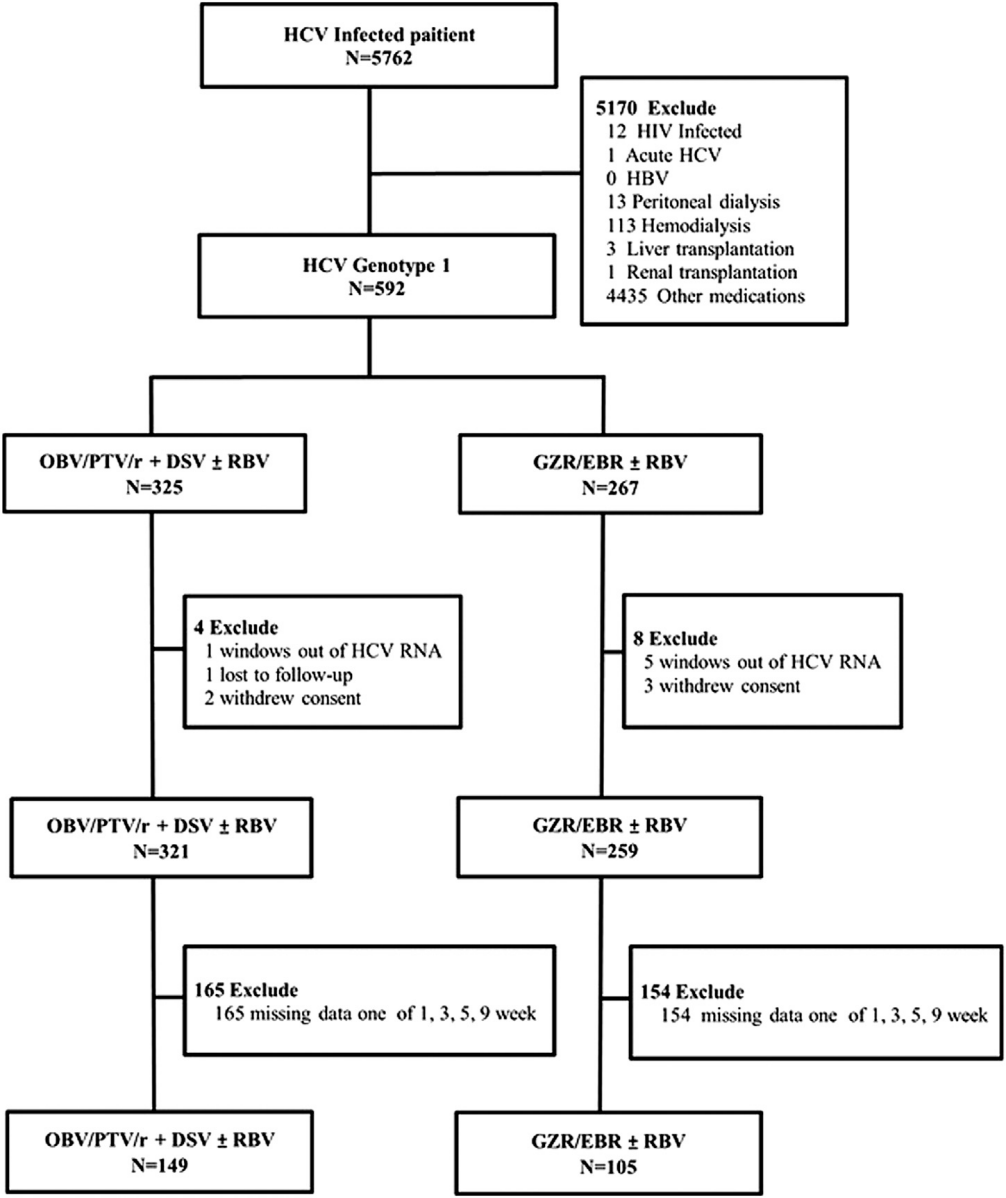
Trial profile.

A total of 5,762 patients were diagnosed with chronic hepatitis C (CHC) between April 1, 2016, and August 31, 2018. Four patients were excluded from the OBV/PTV/r + DSV group: one patient had no measurement of HCV RNA before treatment, one patient had no SVR12 data, and two patients stopped treatment voluntarily because of personal reasons. Eight patients were excluded from the EBR/GZR group: five patients had no measurement of HCV RNA before treatment, and three patients lacked the SVR rate. Of them, 254 eligible patients [average age, 65.51 (SD 10.97) years; 42.91% men and 57.09% women] were included in the final analysis: 149 in the OBV/PTV/r + DSV ± RBV group and 105 in the EBR/GZR ± RBV group; all participants completed the duration treatment (Figure 1). There was no significant between-group differences regarding age and gender. In the OBV/PTV/r + DSV ± RBV group, eight (5.37%) patients had HCV GT 1a infection and the remaining patients had HCV GT 1b infection. However, all patients in the EBR/GZR ± RBV group had HCV GT 1b infection. Furthermore, T-Bil levels and INR were significantly different between the groups (*p* < 0.001).

In terms of complications, 86 patients (57.72%) in the OBV/PTV/r + DSV ± RBV group and 55 patients (52.38%) in the EBR/GZR ± RBV group had peptic ulcer disease (*p* = 0.40). Moreover, overall, there were 33 patients (12.99%) with constipation, 30 (11.81%) with hyperlipidemia, 73 (28.74%) with hypertension, 35 (13.78%) with type 2 diabetes mellitus, 24 (9.45%) with anxiety disorder, and 24 (9.45%) with sleep disorder; however, there were no significant between-group differences regarding the above characteristics (Table 1). In total, 22 patients (14.77%) in the OBV/PTV/r + DSV ± RBV group and 28 (26.67%) in the EBR/GZR ± RBV group received pantoprazole (*p* = 0.019). There were significant between-group differences regarding the administration of lorazepam and sennosides.

**TABLE 1:** Study participants’ baseline and demographic characteristics.

	Total		OBV/PTV/r + DSV ± RBV		EBR/GZR ± RBV		p-Value
	(N = 254)		(*n* = 149)		(*n* = 105)		
Age, mean (SD)							
—	65.51	(10.97)	65.53	(10.41)	65.48	(11.77)	0.17
Gender, n (%)							
Male	109	(42.91)	63	(42.28)	46	(43.81)	0.90
Female	145	(57.09)	86	(57.72)	59	(56.19)	0.90
HCV genotype, n (%)							
1a	8	(3.15)	8	(5.37)	0	(0)	0.02^a^
1b	246	(96.85)	141	(94.63)	105	(100)	0.02^a^
Cirrhosis, n (%)	99	(38.98)	62	(41.61)	37	(35.24)	0.36
Tumor, n (%)	26	(10.24)	15	(10.07)	11	(10.48)	0.92
HCC, n (%)	21	(8.27)	16	(10.74)	5	(4.76)	0.11
HCV RNA, log10IU/mL mean (SD)							
—	6.42	(6.55)	6.45	(6.56)	6.37	(6.54)	0.53
ALT, IU/L (SD)	81.63	(68.56)	86.85	(73.04)	74.21	(61.22)	0.06
AST, IU/L (SD)	73.07	(53.37)	77.95	(55.42)	66.15	(49.77)	0.24
T-Bil, mg/dL (SD)	0.79	(0.39)	0.85	(0.43)	0.71	(0.29)	<0.001
INR	1.06	(0.16)	1.08	(0.19)	1.05	(0.09)	<0.0001
Therapy duration at weeks, n (%)							0.22
8	2	(0.79)	2	(1.34)	0	(0)	—
11	3	(1.18)	2	(1.34)	1	(0.95)	—
12	245	(96.46)	141	(94.63)	104	(99.05)	—
24	4	(1.57)	4	(2.68)	0	(0)	—
RBV combined	15	(5.91)	10	(6.71)	5	(4.76)	0.60
Prior treatment experience, n (%)							
Native	225	(88.58)	131	(87.92)	94	(89.52)	0.84
RBV-base	29	(11.42)	18	(12.08)	11	(10.48)	0.69
IFN-base	16	(6.3)	9	(6.04)	7	(6.67)	0.84
Chronic kidney disease (CKD), n (%)							
Stage 1	154	(60.63)	88	(59.06)	66	(62.86)	0.60
Stage 2 (mild)	66	(25.98)	40	(26.85)	26	(39.39)	0.77
Stage 3 (moderate)	28	(11.02)	20	(13.42)	8	(7.62)	0.16
Stage 4 (severe)	3	(1.18)	1	(0.67)	2	(1.9)	0.57
Stage 5	3	(1.18)	0	(0)	3	(2.86)	0.07
Complications, n (%)							
Peptic ulcer disease	141	(55.51)	86	(57.72)	55	(52.38)	0.40
Gastroesophageal reflux disease	27	(10.63)	10	(6.71)	17	(16.19)	0.016^a^
Constipation	33	(12.99)	20	(13.42)	13	(12.38)	0.81
Hyperlipidemia	30	(11.81)	17	(11.41)	13	(12.38)	0.81
Hypertension	73	(28.74)	43	(28.86)	30	(28.57)	0.96
Type 2 diabetes mellitus	35	(13.78)	21	(14.09)	14	(13.33)	0.86
Anxiety disorder	24	(9.45)	18	(12.08)	6	(5.71)	0.09
Sleep disorder	24	(9.45)	16	(10.74)	8	(7.62)	0.40
Medication for 6 months before starting treatment, n (%)							
Silymarin	185	(72.83)	112	(75.17)	73	(69.52)	0.32
Famotidine 20 mg	113	(44.49)	67	(44.97)	46	(43.81)	0.86
Pantoprazole 40 mg	50	(19.69)	22	(14.77)	28	(26.67)	0.019^a^
Mosapride citrate 5 mg	44	(17.32)	20	(13.42)	24	(22.86)	0.05
Magnesium hydroxide 324 mg	42	(16.54)	28	(18.79)	14	(13.33)	0.25
Dimethylpolysiloxane 40 mg	36	(14.17)	21	(14.09)	15	(14.29)	0.97
Amlodipine 5 mg	32	(12.60)	20	(13.42)	12	(11.43)	0.64
Acetaminophen 500 mg	32	(12.60)	14	(9.40)	18	(17.14)	0.07
Alprazolam 0.5 mg	32	(12.60)	22	(14.77)	10	(9.52)	0.22
Lorazepam 1 mg	29	(11.42)	22	(14.77)	7	(6.67)	0.046^a^
Lansoprazole 15 mg	26	(10.24)	11	(7.38)	15	(14.29)	0.07
Sennosides 20 mg	23	(9.06)	9	(6.04)	14	(13.33)	0.046^a^
Sulpiride 50 mg	22	(8.66)	16	(10.74)	6	(5.71)	0.16

SD, standard deviation; OBV/PTV/r, ombitasvir/paritaprevir/ritonavir; DSV, dasabuvir; IFN, interferon; RBV, ribavirin; EBR/GZR, elbasvir/grazoprevir; ALT, alanine aminotransferase; AST, aspartate aminotransferase; T-Bil, total bilirubin; INR, international normalized ratio.

a Significant difference (*p* < 0.05).

**FIGURE 2:**
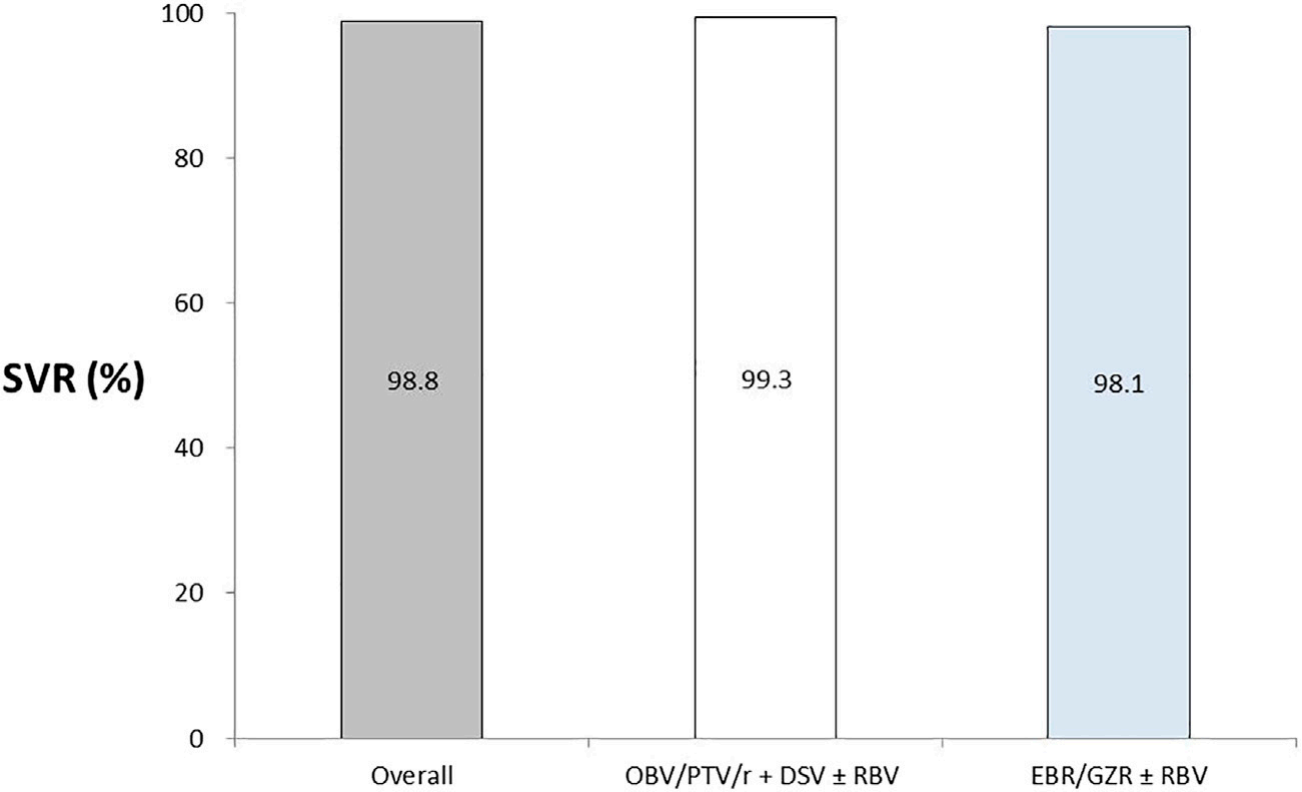
Patients with HCV RNA

Overall, 98.82% of the patients with HCV achieved SVR12; the proportions of patients achieving SVR12 were 99.33% (95% CI, 98.0–100.00) and 98.1% (95% CI, 95.44–100.00) in the OBV/PTV/r + DSV ± RBV and EBR/GZR ± RBV groups, respectively (Figure 2). One patient (0.67%) in the OBV/PTV/r + DSV group and 2 (1.9%) in the EBR/GZR ± RBV group experienced relapse. After treatment, HCV RNA levels markedly decreased to LLOQ at week 2, but one patient experienced relapse in the EBR/GZR ± RBV group at week 18 (Table 2).

**TABLE 2:** Virological response.

Characteristics	Overall			OBV/PTV/r + DSV ± RBV			EBR/GZR ± RBV		
	(N = 254)			(*n* = 149)			(*n* = 105)		
	N	%	(95% CI)	N	%	(95% CI)	N	%	(95% CI)
SVR 12	251	98.82	(97.48–100.16)	148	99.33	(98.02–100.00)	103	98.1	(95.44–100.00)
Relapse	3	1.18	(0.16–2.52)	1	0.67	(0.65–0.20)	2	1.9	(0.75–4.56)

OBV/PTV/r, ombitasvir/paritaprevir/ritonavir; DSV, dasabuvir; RBV, ribavirin; EBR/GZR, elbasvir/grazoprevir; SVR12, sustained virologic response after treatment 12 weeks; 95% CI, confidence interval.

Among the patients with HCV treated with OBV/PTV/r and EBR/GZR, nine patients (3.54%) developed DILI as determined during follow-up care. Of these nine patients, three (2.01%) received the OBV/PTV/r + DSV ± RBV regimen and six (5.71%) received the EBR/GZR ± RBV regimen. The IRR of DILI caused by using EBR/GZR was 2.84 times (0.71–11.35) higher than that caused by using OBV/PTV/r + DSV; however, the difference was not significant (*p* = 0.14).

In the OBV/PTV/r + DSV ± RBV group, there were two patients (1.34%) with ALT >5 times ULN, one patient (0.67%) with AST >5 times ULN, two patients (1.34%) with ALT or AST >3 times ULN combined with T-Bil > 2 times ULN and with ALT >5 times ULN, but none with ALT or AST >3 times ULN combined with INR >1.5 and T-Bil > 2 times ULN combined with INR >1.5. In the EBR/GZR ± RBV group, there were four patients (3.81%) with ALT >5 times ULN, two patients (1.90%) with AST >5 times ULN, one patient (0.95%) with ALT or AST >3 times ULN combined with T-Bil > 2 ULN and with ALT >5 times ULN, but none with ALT or AST >3 times ULN combined with INR >1.5 and T-Bil > 2 times ULN combined with INR >1.5.

**FIGURE 3:**
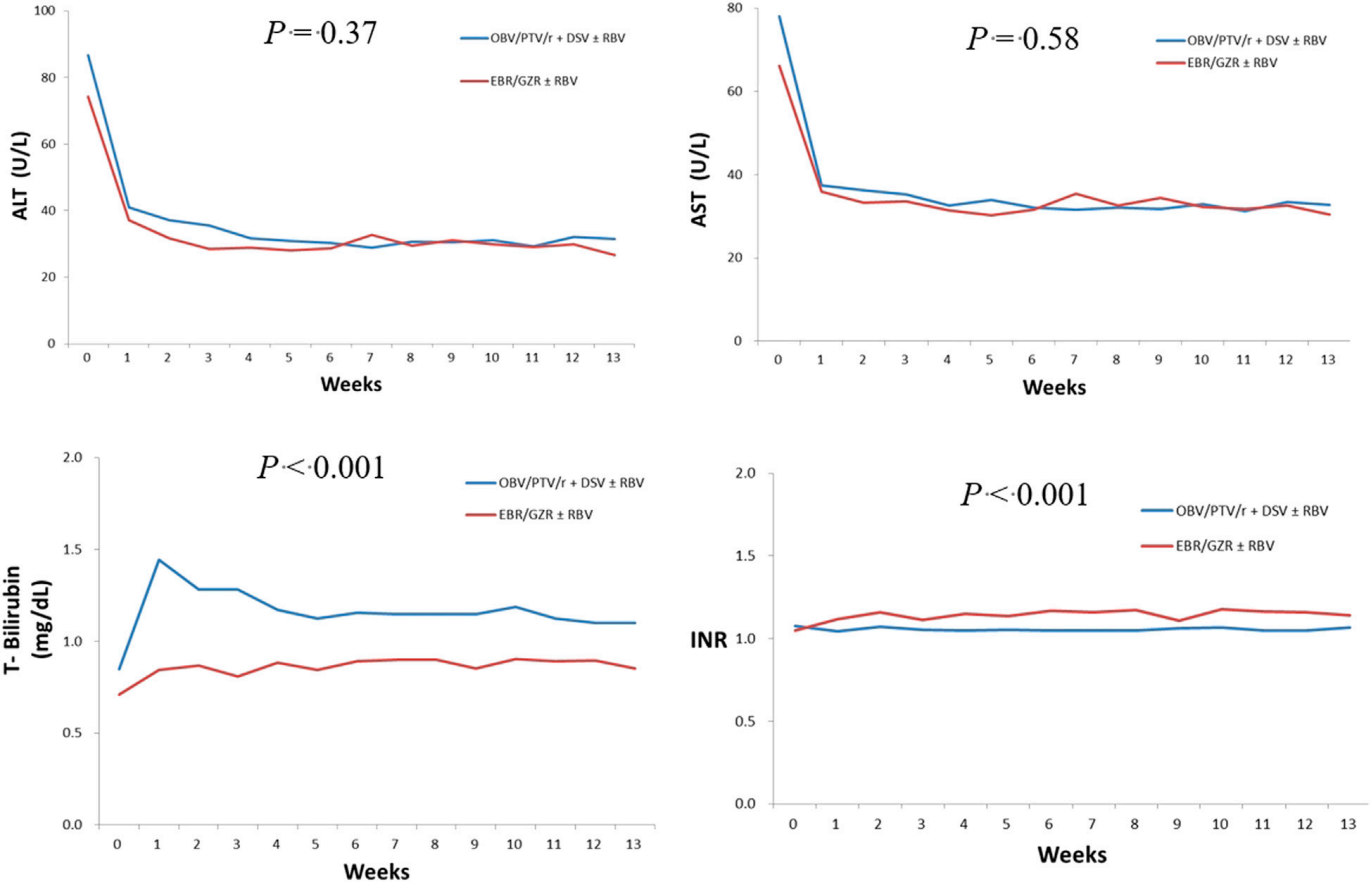
Laboratory assessments.

The IRR of ALT >5 times ULN, AST >5 times ULN and ALT or AST >3 times ULN combined with T-Bil > 2 times ULN were 2.84 times (*p* = 0.23), 2.84 times (*p* = 0.39), and 0.71 times (*p* = 0.78) higher, respectively, with EBR/GZR use than with OBV/PTV/r + DSV use; however, there was no significant between-group difference (Table 3). After the administration of OBV/PTV/r + DSV and EBR/GZR, we observed changes in ALT, AST, and T-Bil levels and INR at 12 weeks of treatment. There were no significant between-group differences regarding ALT and AST levels (*p* = 0.37 and 0.58, respectively); however, INR and T-Bil levels were significantly higher in the OBV/PTV/r + DSV group (*p* < 0.001 for both) (Figure 3).

**TABLE 3:** Incidence of the drug-induced liver injury adverse events.

	OBV/PTV/r + DSV ± RBV	EBR/GZR ± RBV	Overall	IRR	(95% CI)	p-Value
Event, n (%)	3 (2.01)	6 (5.71)	9 (3.54)	2.84	(0.71–11.35)	0.14
ALT >5×ULN	2 (1.34)	4 (3.81)	6 (2.36)	2.84	(0.52–15.49)	0.23
AST >5×ULN	1 (0.67)	2 (1.90)	3 (1.18)	2.84	(0.26–31.30)	0.39
ALT/AST >3×ULN + T-Bil >2×ULN^a^	2 (1.34)	1 (0.95)	3 (1.18)	0.71	(0.06–7.82)	0.78
ALT/AST >3×ULN + INR >1.5	0	0	0	0	−	−
T-Bil >2×ULN + INR >1.5	0	0	0	0	−	−

OBV/PTV/r, ombitasvir/paritaprevir/ritonavir; DSV, dasabuvir; RBV, ribavirin; EBR/GZR, elbasvir/grazoprevir; ALT, alanine aminotransferase; AST, aspartate aminotransferase; T-Bil, total bilirubin; INR, international normalized ratio; IRR, incidence rate ratio; CI, confidence interval; ULN, upper limit of normal.

a Including the patients with ALT> 5 times ULN.

We observed changes in ALT, AST, and T-Bil levels and INR in patients who developed DILI after 12 weeks of treatment with OBV/PTV/r + DSV or EBR/GZR. The elevated ALT levels in the OBV/PTV/r + DSV ± RBV group were >5 times ULN after drug administration at week 3 and >3 times ULN at weeks 5, 9, and 12 consecutively. We also detected that the elevated ALT level in the EBR/GZR ± RBV group was >3 times ULN at weeks 7 and 9.

**FIGURE 4:**
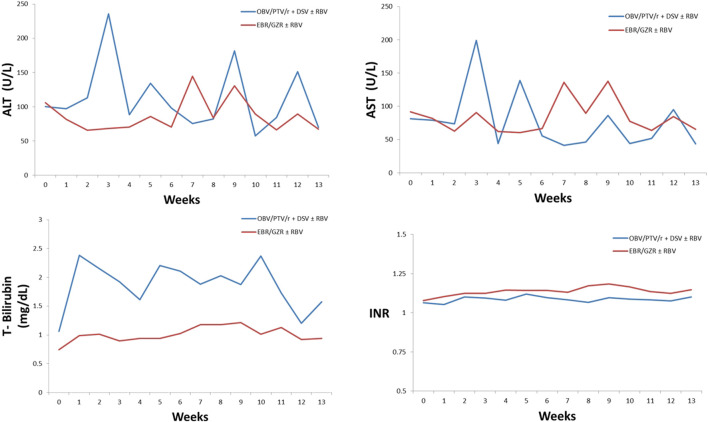
Laboratory parameters among patients with DILI adverse events.

The increased AST level in the OBV/PTV/r + DSV ± RBV group was >5 times ULN at weeks 3 and 9 and >3 times ULN at weeks 5 and 12. In the EBR/GZR ± RBV group, AST increased by > 3 times ULN at weeks 3, 7, 9, and 12. In the OBV/PTV/r + DSV ± RBV group, two patients experienced T-Bil levels elevated to >2 times ULN after drug administration at week 5. None of the patients developing DILI AEs among those receiving OBV/PTV/r + DSV and EBR/GZR had an INR >1.5 during the 12-week treatment period (Figure 4).

As determined *via* the sensitivity analysis (Table 4), overall, a total of 18 patients (7.09%) developed DILI. Among these patients, 11 were in the OBV/PTV/r + DSV ± RBV group and 7 (6.67%) in the EBR/GZR ± RBV group. The same outcome was also observed as an original criterion for liver injury.

**TABLE 4:** Sensitivity analysis—the change in the ALT/AST standard.

	OBV/PTV/r + DSV ± RBV	EBR/GZR ± RBV	Overall	IRR	(95% CI)	p-Value
Event, n (%)	11 (7.38)	7 (6.67)	18 (7.09)	0.90	(0.35–2.33)	0.83
ALT >3 times ULN	10 (6.71)	5 (4.76)	15 (5.91)	0.90	(0.35–2.33)	0.83
AST >3 times ULN	8 (5.37)	4 (3.81)	12 (4.72)	0.71	(0.24–2.08)	0.53
ALT/AST >3 times ULN + T-Bil >2 times ULN^a^	2 (1.34)	1 (0.95)	3 (1.18)	0.71	(0.06–7.82)	0.78
ALT/AST >3 times ULN + INR >1.5	0	0	0	0	−	−
T-Bil >2 times ULN + INR >1.5	0	0	0	0	−	−

OBV/PTV/r, ombitasvir/paritaprevir/ritonavir; DSV, dasabuvir; RBV, ribavirin; EBR/GZR, elbasvir/grazoprevir; ALT, alanine aminotransferase; AST, aspartate aminotransferase; T-Bil, total bilirubin; INR, international normalized ratio; IRR, incidence rate ratio; CI, confidence interval; ULN, upper limit of normal.

a Including the patients with ALT> 5 times ULN.

In Subgroup analysis that relate risks among SVR12, Relapse, and DILI event in Supplementary Table S1. There have more frequency induced the risk of DILI events, whom have liver tumors, HCC, Hypertension, and mild to severe CKD, and patients with HCV age between 55–74 years. Whereas both regimens combined with RBV, it no increment in incidence of DILI. Beside of that adverse event of DILI had occurred during treatment, which rarely affect the risk of recurrence or SVR.

In OBV/PTV/r + DSV group, the majority of DILI events were observed in men, and the age range was 55–84. On the other hand, the DILI AEs in the EBR/GZR group accounted for 50% of men and women, of which two cases were in the 55–64 age group, three cases in 65–74, and one case over 85.

## Discussion

This study provides real-world evidence of the efficacy, in terms of antiviral potency, of OBV/PTV/r + DSV and EBR/GZR treatment for CHC, including patients with HCV GT 1a infection and severe chronic renal failure. Overall, 98.82% patients achieved SVR12. The relapse rates in the OBV/PTV/r + DSV ± RBV and EBR/GZR ± RBV groups were 0.67% (1/149) and 1.9% (2/105), respectively.

EBR/GZR for patients with HCV who were treated with interferon and RBV resulted in a lower SVR12 rate of 90.91% (10/18), which is similar to the results of a previous study, wherein patients with HCV GT 1, 4, or 6 infection were divided into the EBR/GZR and EBR/GZR + RBV groups during 12 weeks of treatment; SVR12 rates in the two groups were 88.9% (48/54) and 91.4% (74/81), respectively (Jacobson et al., 2017). However, the result reported on here do not support the findings of previous research on hard-to-treat patients who were prior DAA exposed with virological failure, the majority of patients with cirrhosis or severe fibrosis, the frequent presence of NS5A and NS3 resistance-associated variants (RAVs) at baseline, these had received EBR/GZR and SVR12 was achieved 96% (Buti et al., 2016; de Lédinghen et al., 2018).

Furthermore, a prospective and randomized study showed that EBR/GZR ± RBV was highly efficacious in inducing SVR12 in patients with HCV GT 1, 4, or 6 infection which failed on previous treatment with peg-interferon and RBV, PI-based combination regimens, NS3 RAVs, and cirrhosis and/or a prior null response (Buti et al., 2016; Kwo et al., 2017). Moreover, data from the present study confirm high rates of SVR12 and SVR24 (94.2 and 94.6%, respectively) in the Asia-Pacific region and Russia (Wei et al., 2019) and highly efficacious, well tolerated in cirrhotic and noncirrhotic Japanese patients with HCV infection who received EBR/GZR for 12 weeks (Kumada et al., 2017). In addition, GZR for 100 and 50 mg were similarly effective, with SVR rates of 96.8 and 100% respectively (Kumada et al., 2017).

Several studies described that there were 3 relapses occurring by post-therapy week 8 whom baseline RAVs stably reappeared at relapse and persisted throughout for the full 24-week follow-up period (Buti et al., 2016). Also, the relapse rate for EBR/GZR was 1.4% in meta-analysis (Ahmed et al., 2018). The OBV/PTV/r virologic relapse rate of GT1 patients was 1.3% (Wedemeyer et al., 2017).

Our results confirm that SVR12 achieved by our patients with HCV GT 1a infection treated with OBV/PTV/r + DSV ± RBV was same as that achieved by those with severe chronic renal failure treated with EBR/GZR. The overall SVR12 rate among patients infected with HCV GT 1a was 93.8% (95% CI, 87.8–98.0) (Flisiak et al., 2016). Furthermore, among patients with severe renal insufficiency (stage 4 or 5 chronic kidney disease, including those under hemodialysis) and compensatory liver disease (with or without cirrhosis), the SVR12 rate after EBR/GZR therapy for HCV GT 1 infection was approximately 99% (115/116; 95% CI, 95.3–100.0) (Roth et al., 2015).

These outcomes, which are consistent with the addition of RBV, did not significantly increase the efficacy of EBR/GZR combination in HCV GT 1 infection. For cirrhotic patients, the SVR rate was 95.7%, and for non-cirrhotic patients, the SVR rate was 97%. However, this regimen achieved lower SVR rates (<90%) in patients with NS5A RAS (Ahmed et al., 2018). 16 or 24 weeks of combination of sofosbuvir + EBR/GZR + RBV SVR is 100% (de Lédinghen et al., 2018).

The excluded population of this study that HCV GT 1 mono-infection or HIV/HCV co-infection with oral once-daily EBR/GZR ± RBV in previously untreated patients without cirrhosis. The mono-infected and co-infected patients treated without RBV were 98 and 87%, respectively, and with RBV were 93 and 97%, respectively (Sulkowski et al., 2015). For both cirrhotic and without cirrhotic patients and treatment-experienced patients or those with NS3 RAS or HCV/HIV co-infection can be treated successfully in 12 weeks (Ahmed et al., 2018). Additionally, participants with HCV GT 2 infection received GZR 100 mg + RBV ± EBR 50 mg, and those with GT 4, 5, or 6 infection were randomized to receive EBR/GZR ± RBV, all for 12 weeks. Among the GT 2 population, SVR12 rates were slightly higher in participants receiving EBR/GZR + RBV compared with participants receiving GZR + RBV (80 vs. 73%). SVR rates were higher in participants with HCV GT 4 infection. In contrast, EBR/GZR + RBV appeared to increase SVR12 in HCV GT 5 infections. In participants with GT 6 infection, SVR12 was 75% in those receiving EBR/GZR and EBR/GZR + RBV (Brown et al., 2018).

In an indirect comparative network integration analysis study evaluating the relative safety results of various treatment prescriptions for CHC without interferon, the results showed a significantly lower incidence of AEs in the EBR/GZR group than in the OBV/PTV/r + DSV + RBV group, and the odds ratio was 4.09 (95% CI, 1.17–14.09) (Ferreira et al., 2016). The pooled RR showed no significant difference between EBR/GZR and EBR/GZR + RBV in terms of serious AEs (RR = 1.19; 95% CI, 0.29–4.80; *p* = 0.65) (Ahmed et al., 2018). On the other hand, the safety profile of EBR/GZR ± RBV was similar in mono-infected and co-infected patients. No patient died or discontinued due to an adverse event, laboratory abnormality (Buti et al., 2016), virological failure (de Lédinghen et al., 2018), or had ALT or AST values that met the criteria for late ALT or AST level elevation (Kumada et al., 2017). However, 1.7% of patients discontinued due to AEs, most often in the treatment arm that received 16 weeks of treatment unrelated to ALT elevation (Kwo et al., 2017). The results were in agreement with no significant difference between the EBR/GZR group and EBR/GZR + RBV group in terms of serious AEs, headache, fatigue, lower Hb levels (<8.5 g/dl) on treatment, ALT elevation (>2.5x baseline) on treatment, and AST elevation (>2.5x baseline) on treatment. All safety comparisons and pooled studies were homogeneous,^1113^ and also, all ALT elevations returned to baseline after the study medication was discontinued and all subjects with an ALT elevation >5x ULN achieved SVR (Kwo et al., 2017). Also, tolerability was similar in both GZR doses of 50 and 100 mg, with a comparable incidence of drug-related AEs (32.3 vs. 29.0%) (Kumada et al., 2017).

Sudden ALT elevation during DAA therapy is an unusual but noticeable AE in CHC patients, which may result in early termination of treatment (Liu et al., 2020). Regarding safety outcomes, the IRR of DILI AEs caused by EBR/GZR was 2.84 times (0.71–11.35) higher than that caused by OBV/PTV/r + DSV (*p* = 0.14), indicating no significant between-group differences. It is known that patients with HCV with liver tumors or mild-to-severe chronic renal failures have a high risk of developing DILI. The results were in agreement with the results of a previous clinical trial: ALT elevation (>2.5 times the baseline) on treatment (RR = 1.24; 95% CI, 0.07–9.76; *p* = 0.88) and AST elevation (>2.5 times the baseline) on treatment (RR = 1.24; 95% CI, 0.07–19.76; *p* = 0.88) (Ahmed et al., 2018). Similarly, 1.0% of patients had late elevations of ALT or AST (>5 X ULN), but these elevations were transient and did not require interruption or discontinuation of EBR/GZR (Kwo et al., 2017).

In our study, DILI occurred in the OBV/PTV/r + DSV group, in which the male:female ratio was 2:1 and age range was 55–84 years; the male:female ratio was similar in the EBR/GZR group with the age range of 55–74 years. DILI in these patients was classified as moderate (ALT ≥5 times ULN or ALP ≥2 times ULN and T-Bil ≥ 2 times ULN or INR ≥1.5) (European Association for, 2019). The Clinical Practice Guidelines (2019) of the European Association for the Study of the Liver indicate that chronic HBV or HCV infection and alcoholic liver disease are the risk factors for DILI and that ALT or AST levels might increase in the affected patients due to the nature of these diseases. Age (>55 years) and gender (especially women) are also considered risk factors for DILI. The incidence rate of adverse drug reactions increases with age due to altered drug clearance. The guidelines also indicate that women are more likely to develop primary biliary cholangitis and autoimmune hepatitis (European Association for, 2019). The incidence of drug-related AEs, discontinuations owing to an adverse event, and abnormal elevations of ALT, AST, and bilirubin were similar in younger (≤35 years) and older (>35 years) populations (Asselah et al., 2020).

The product manual of the drug indicates that ALT levels in the OBV/PTV/r + DSV ± RBV group increased by > 5 times ULN during the first 4 weeks of the treatment period and decreased at weeks 2–8 following continuous administration (Product Information, 2015). Reportedly, ALT levels in the EBR/GZR ± RBV group were five times ULN at week 8, and the levels of most parameters decreased under continuous treatment or by the end of treatment (Product Information, 2016). In previous studies, once daily EBR/GZR for 12 weeks, sole patient who died, but HCV RNA was negative at 5 weeks after stopping treatment (de Lédinghen et al., 2018). In another study, one patient receiving GZR plus RBV also had a late elevation in ALT/AST ≤5X ULN at or after therapy at week 4 (Brown et al., 2018). Moreover, late ALT or AST elevations >5× ULN were reported in 1.1% of participants in the immediate-treatment group and 2.5% of participants receiving active treatment in the deferred-treatment group (Wei et al., 2019). However, no patient had an ALT increase to 5X UNL at or after 4 weeks of therapy (Buti et al., 2016). On observing patients with DILI, it was found that ALT levels were elevated after the administration of the regimen, which is different from real-world data. Additionally, ALT levels were decreased when the treatment was continued. The rise in ALT levels did not affect the cure rate. This finding also supports that on-treatment ALT elevation occurred in CHC patients treated with preferred DAAs, but had no impact on SVR (Liu et al., 2020).

It is known that patients with HCV with liver tumors or mild-to-severe chronic renal failures have a high risk of developing DILI. However, the risk of DILI does not increase with the addition of RBV to the treatment regimen, and the incidence of DILI is not associated with relapse. These results lend some credence to the hypothesis that the addition of RBV to the treatment regimen which is generally utilized as an adjuvant drug in several HCV treatment regimens reduces viral relapse risk (Ahmed et al., 2018). In contrast, the frequency of drug-related AEs, bilirubin elevations, or hemoglobin decreases was higher in the regimens EBR/GZR + RBV than in those without RBV.^917^

In this large-scale real-world study, the incidence rate of on-treatment ALT elevation and ≥grade 3 ALT elevation was 10.9 and 1.4%, respectively, under currently recommended DAAs. Higher pretherapy ALT and HBV coinfection were the risk factors for on-treatment ALT elevation during preferred DAAs treatment, which had no impact on SVR rates, and only one patient had early terminated treatment but still achieved SVR. To our knowledge, this is the first real-world study addressing not only the incidence but also the time of onset, predictors, and clinical impact of on-treatment ALT elevation among different DAAs (Liu et al., 2020).

The frequency of “on-treatment ALT elevation” was the highest in those treated with EBR/GZR (12.3%), followed by sofosbuvir-based regimen (11.6%) and the least in G/P (5.4%) treated patients, similar to those treated with OBV/PTV/r + DSV (10.8%), but much lower than that during asunaprevir/daclatasvir (ASV/DCV) (39.9%). Among patients treated with preferred DAAs, cirrhosis, HBV coinfection, BMI ≥25, HbA1c ≥ 6.5, HOMA index ≥2, triglyceride ≥150 mg/dl, the use of sofosbuvir-based or EBR/GZR regimens, pre-therapy ALT ≥1xULN, higher AST, AFP, T-Bil, and lower albumin level was found (Liu et al., 2020). Among 7, 3.2, 1.9 and 1% of patients with ASV/DCV, EBR/GZR, OBV/PTV/r + DSV, and sofosbuvir-based regimen had ALT elevation ≥ grade 3, respectively. The events of T-Bil elevation were observed in 13.2% patients treated with preferred DAA, highest in those treated by sofosbuvir-based regimen (16.4%) followed by G/P (8.5%) and EBR/GZR (7.8%), and much lower than those treated with ASV/DCV (23.1%) and OBV/PTV/r + DSV (29.4%). Grade 3/4 abnormality occurred mainly in patients with OBV/PTV/r + DSV (2.5%), followed by sofosbuvir-based (1.2%), G/P (0.9%), ASV/DCV (0.7%), and none with EBR/GZR (Liu et al., 2020).

OBV/PTV/r + DSV and EBR/GZR can be used to treat HCV infection, decrease the elevated ALT and ALT levels to the normal range and also play an essential role as DAAs. Compared with previous therapeutic agents, DAAs like OBV/PTV/r + DSV and EBR/GZR do not cause irreversible hepatic injury and can reverse DILI and high inflammation index caused by continuous treatment without discontinuing therapy immediately. However, with the development of new DAA agents for HCV infections, OBV/PTV/r + DSV has been replaced gradually. Nowadays, the AASLD-IDSA HCV guidance for genotype 1 HCV treatment of patients in whom prior therapy failed or treatment-naïve and with or without compensated cirrhosis, the EBR/GZR for 12 weeks is recommended for first-line therapy (Class I, Level A). Moreover, the unique role of EBR/GZR is one of the preferred treatment choices for HCV combined with CKD (AASLD).

There are some limitations in this study. First, the major conditions are the small sample size and restricted statistical power. Second, the target population of the study included patients with HCV GT 1 infection. However, only 8 patients with HCV GT 1a infection were identified; the remaining patients were infected with HCV GT 1b. Third, because of the retrospective nature of the study, liver-associated test items could not be fully included, such as hemoglobin, γ-glutamyl transpeptidase, α-fetoprotein, and alkaline phosphatase; thus, we could not use the values of ALP and ALT to calculate the R value to further assess the type of liver injury. Fourth, during the implementation of this study, patients treated with available DAA regimen were limited due to glecaprevir/pibrentasvir and velpatasvir-based regimens that have not got licenses. Finally, the limitation due to use of hospital medical records also led to the inability to access additional information, such as smoking status, life style, and social status. Thus, risk factors could not be illustrated in detail.

## Conclusion

In conclusion, HCV infection is a risk factor for DILI, and DILI occurrence during OBV/PTV/r + DSV or EBR/GZR treatment should be considered while using hepatotoxic drugs, which induce AEs associated with liver injury; however, these events subside afterward upon continuation and completion of treatment regimen without impacting the cure rate. The present study results were based on real-world data that could apply to real-world patients. Inclusion of laboratory data could provide more definitive results; they provide us with additional clinical therapeutic options.

## Data Availability

The original contributions presented in the study are included in the article/Supplementary Material, and further inquiries can be directed to the corresponding author.
