# Supplementary material for: A Retrospective Cohort Study: Safety and Effectiveness of Elbasvir/Grazoprevir ± Ribavirin Compared With Ombitasvir/Paritaprevir/Ritonavir/Dasabuvir ± Ribavirin in Patients With Chronic Hepatitis C Genotype 1 Infection
Source: Front Pharmacol. 2021 Sep 9;12:640317. doi: 10.3389/fphar.2021.640317 (PMC8458878; doi:10.3389/fphar.2021.640317)
Supplement: Supplementary file 1 [file Table1.docx]

Table S1. Subgroup analysis—relate risks among SVR12, Relapse, and DILI event.

|  | SVR12 | | | | | Relapse | | | | | | DILI event | | | | | |
| --- | --- | --- | --- | --- | --- | --- | --- | --- | --- | --- | --- | --- | --- | --- | --- | --- | --- |
|  | OBV/PTV/r + DSV ±RBV | | EBR/GZR ± RBV | | RR |  | OBV/PTV/r + DSV ±RBV | | EBR/GZR ± RBV | | RR |  | OBV/PTV/r + DSV ±RBV | | EBR/GZR ± RBV | | RR |
|  | (N =148) | | (N =103) | |  |  | (N =1) | | (N =2) | |  |  | (N =3) | | (N =6) | |  |
|  | N | SVR12 (%) | N | SVR12 (%) |  |  | N | (Incidence %) | N | (Incidence %) |  |  | N | (Incidence %) | N | (Incidence %) |  |
| Event | 3 | (100.00) | 6 | (100.00) | 0.11 | Event | 0 | (0) | 0 | (0) | 0.11 | Relapse | 0 | (0) | 0 | (0) | 0.11 |
| Age |  |  |  |  |  |  |  |  |  |  |  |  |  |  |  |  |  |
| <44 | 6 | (100.00) | 5 | (100.00) | - |  | 0 | (0) | 0 | (0) | - |  | 0 | (0) | 0 | (0) | - |
| 45-54 | 17 | (100.00) | 15 | (88.24) | 2.13 |  | 0 | (0) | 2 | (13.33) | 2.14 |  | 0 | (0) | 0 | (0) | - |
| 55-64 | 47 | (97.92) | 18 | (100.00) | 0.38 |  | 1 | (2.08) | 0 | (0) | 0.38 |  | 1 | (2.08) | 2 | (11.11) | 2.46 |
| 65-74 | 47 | (100.00) | 46 | (100.00) | - |  | 0 | (0) | 0 | (0) | - |  | 1 | (2.13) | 3 | (6.52) | 1.09 |
| 75-84 | 28 | (100.00) | 14 | (100.00) | - |  | 0 | (0) | 0 | (0) | - |  | 1 | (3.57) | 0 | (0) | 0.51 |
| ≧85 | 3 | (100.00) | 4 | (100.00) | - |  | 0 | (0) | 0 | (0) | - |  | 0 | (0) | 1 | (25.00) | 0.88 |
| Gender |  |  |  |  |  |  |  |  |  |  |  |  |  |  |  |  |  |
| Male | 62 | (98.41) | 46 | (100.00) | 0.11 |  | 1 | (1.59) | 0 | (0) | 0.11 |  | 2 | (3.17) | 3 | (5.08) | 0.61 |
| Female | 86 | (100.00) | 57 | (96.61) | 0.11 |  | 0 | (0) | 2 | (3.39) | 0.11 |  | 1 | (1.16) | 3 | (5.08) | 0.61 |
| HCV genotype 1a | 8 | (100.00) | 0 | (0) | 0.1 |  | 0 | (0) | 0 | (0) | 0.1 |  | 0 | (0) | 0 | (0) | 0.3 |
| HCV genotype 1b | 140 | (99.29) | 103 | (98.10) | 0.1 |  | 1 | (0.71) | 2 | (1.90) | 0.1 |  | 3 | (2.13) | 6 | (5.71) | 0.3 |
| RBＶCombined | 10 | (100.00) | 5 | (100.00) | 0.19 |  | 0 | (0) | 0 | (0) | 0.19 |  | 0 | (0) | 0 | (0) | 0.59 |
| Cirrhosis | 61 | (98.39) | 37 | (100.00) | 0.04 |  | 1 | (1.61) | 0 | (0) | 0.04 |  | 1 | (1.61) | 3 | (8.11) | 0.12 |
| Toumer | 15 | (100.00) | 11 | (100.00) | 0.35 |  | 0 | (0) | 0 | (0) | 0.35 |  | 1 | (6.67) | 1 | (9.09) | 1.46 |
| HCC | 16 | (100.00) | 5 | (100.00) | 0.27 |  | 0 | (0) | 0 | (0) | 0.27 |  | 1 | (6.25) | 1 | (20.00) | 2.4 |
| Prior treatment response | | | | | | | | | | | | | | | | | |
| Native | 130 | (99.24) | 93 | (98.94) | 1.44 |  | 1 | (0.76) | 1 | (1.06) | 1.44 |  | 3 | (2.29) | 6 | (6.38) | 1.2 |
| RBV-base | 18 | (100.00) | 10 | (90.91) | 1.44 |  | 0 | (0) | 1 | (9.09) | 1.44 |  | 0 | (0) | 0 | (0) | 1.2 |
| IFN-base | 9 | (100.00) | 6 | (85.71) | 3.76 |  | 0 | (0) | 1 | (14.29) | 3.76 |  | 0 | (0) | 0 | (0) | 0.63 |
| Chronic kidney disease (CKD) | | | | | | | | | | | | | | | | | |
| Stage 1 | 88 | (100.00) | 64 | (96.97) | 0.05 |  | 0 | (0) | 2 | (3.03) | 0.05 |  | 1 | (1.14) | 4 | (6.06) | 0.1 |
| Stage 2 (mild) | 39 | (97.50) | 26 | (100.00) | 0.09 |  | 1 | (2.50) | 0 | (0) | 0.09 |  | 0 | (0) | 0 | (0) | 3.28 |
| Stage 3 (moderate) | 19 | (100.00) | 8 | (100.00) | 0.38 |  | 0 | (0) | 0 | (0) | 0.38 |  | 2 | (10.00) | 1 | (12.50) | 4.73 |
| Stage 4 (severe) | 1 | (100.00) | 2 | (100.00) | 0.04 |  | 0 | (0) | 0 | 0.00 | 0.04 |  | 0 | (0) | 1 | (50.00) | 7.88 |
| Stage 5 | 0 | (0) | 3 | (100.00) | 0.04 |  | 0 | (0) | 0 | 0.00 | 0.04 |  | 0 | (0) | 0 | (0) | 0.11 |
| Complications | | | | | | | | | | | | | | | | | |
| Peptic ulcer disease | 85 | (98.84) | 55 | (100.00) | 0.6047 |  | 1 | (1.16) | 0 | (0) | 0.6047 |  | 3 | (3.49) | 3 | (5.45) | 0.4701 |
| Gastroesophageal reflux disease | 10 | (37.04) | 17 | (62.96) | 0.3611 |  | 0 | (0) | 0 | (0) | 0.3611 |  | 0 | (0) | 1 | (5.88) | 0.0023 |
| Constipation | 20 | (60.61) | 13 | (39.39) | 0.4533 |  | 0 | (0) | 0 | (0) | 0.4533 |  | 1 | (5.00) | 1 | (7.69) | 0.7032 |
| Hyperlipidemia | 17 | (56.67) | 13 | (43.33) | 1.3501 |  | 1 | (5.88) | 0 | (0) | 1.3501 |  | 0 | (0) | 1 | (7.69) | 0.0044 |
| Hypertension | 43 | (58.90) | 30 | (41.10) | 0.0313 |  | 0 | (0) | 1 | (3.33) | 0.0313 |  | 0 | (0) | 4 | (13.33) | 1.1236 |
| Type 2 diabetes mellitus | 21 | (60.00) | 14 | (40.00) | 0.4852 |  | 0 | (0) | 0 | (0) | 0.4852 |  | 0 | (0) | 1 | (7.14) | 0.0559 |
| Anxiety disorder | 18 | (75.00) | 6 | (25.00) | 0.3168 |  | 0 | (0) | 0 | (0) | 0.3168 |  | 1 | (5.56) | 0 | (0) | 0.0301 |
| Sleep disorder | 16 | (66.67) | 8 | (33.33) | 0.3168 |  | 0 | (0) | 0 | (0) | 0.3168 |  | 0 | (0) | 0 | (0) | 0.9736 |
| Medication 6 months before starting treatment | | | | | | | | | | | | | | | | | |
| Silymarin | 112 | (60.54) | 73 | (39.46) | 1.1323 |  | 1 | (0.89) | 2 | (2.74) | 1.1323 |  | 2 | (1.79) | 4 | (5.48) | 0.1794 |
| Famotidine 20mg | 67 | (59.29) | 46 | (40.71) | 0.153 |  | 0 | (0) | 1 | (2.17) | 0.153 |  | 2 | (2.99) | 4 | (8.70) | 1.8584 |
| Pantoprazole 40mg | 22 | (44.00) | 28 | (56.00) | 0.3577 |  | 0 | (0) | 1 | (3.57) | 0.3577 |  | 0 | (0) | 0 | (0) | 2.2869 |
| Mosapride citrate 5mg | 20 | (45.45) | 24 | (54.55) | 0.6361 |  | 0 | (0) | 0 | (0) | 0.6361 |  | 0 | (0) | 1 | (4.17) | 0.2514 |
| Magnesium hydroxide 324mg | 28 | (66.67) | 14 | (33.33) | 0.6207 |  | 1 | (3.57) | 0 | (0) | 0.6207 |  | 1 | (3.57) | 2 | (14.29) | 1.9077 |
| Dimethylpolysiloxane 40mg | 21 | (58.33) | 15 | (41.67) | 0.5013 |  | 0 | (0) | 0 | (0) | 0.5013 |  | 0 | (0) | 1 | (6.67) | 0.0719 |
| Amlodipine besylate 5mg | 20 | (62.50) | 12 | (37.50) | 0.4376 |  | 0 | 0.00 | 0 | 0.00 | 0.4376 |  | 0 | (0) | 1 | (8.33) | 0.0187 |
| Acetaminophen 500mg | 14 | (43.75) | 18 | (56.25) | 0.4376 |  | 0 | (0) | 0 | (0) | 0.4376 |  | 0 | (0) | 1 | (5.56) | 0.0187 |
| Alprazolam 0.5mg | 22 | (68.75) | 10 | (31.25) | 1.1854 |  | 1 | (4.55) | 0 | (0) | 1.1854 |  | 0 | (0) | 0 | (0) | 1.345 |
| Lorazepam 1mg | 22 | (75.86) | 7 | (24.14) | 0.3913 |  | 0 | (0) | 0 | (0) | 0.3913 |  | 1 | (4.55) | 0 | (0) | 0.0009 |
| Lansoprazole 15 mg | 11 | (42.31) | 15 | (57.69) | 0.3462 |  | 0 | (0) | 0 | (0) | 0.3462 |  | 0 | (0) | 0 | (0) | 1.064 |
| Sennosides 20mg | 9 | (39.13) | 14 | (60.87) | 0.3023 |  | 0 | (0) | 0 | (0) | 0.3023 |  | 0 | (0) | 1 | (7.14) | 0.0479 |
| Sulpiride 50mg | 16 | (72.73) | 6 | (27.27) | 0.2879 |  | 0 | (0) | 0 | (0) | 0.2879 |  | 1 | (6.25) | 0 | (0) | 0.0708 |
| SD, standard deviation; OBV/PTV/r, ombitasvir/paritaprevir/ritonavir; DSV, dasabuvir; IFN, interferon; RBV, ribavirin; EBR/GZR, elbasvir/grazoprevir; ALT, alanine aminotransferase; AST, aspartate aminotransferase; T-Bil, total bilirubin; INR, international normalised ratio | | | | | | | | | | | | | | | | | |
